# Action scaling of distance perception is task specific and does not predict “the embodiment of culture”: a comment on Soliman, Gibson, and Glenberg (2013)

**DOI:** 10.3389/fpsyg.2014.00302

**Published:** 2014-04-21

**Authors:** Andrew D. Wilson

**Affiliations:** School of Social, Psychological and Communication Sciences, Leeds Metropolitan UniversityLeeds, UK

**Keywords:** Proffitt, embodiment, action-scaling, perceived distance

There is now extensive evidence to support James J. Gibson's (1966, 1979) hypothesis that our perception of the environment is scaled in terms of our ability to act on that environment; by its *affordances*. One strand of evidence comes from Proffitt, who has shown that changing a person's ability to act affects how they judge their ability to perform an upcoming task. The most famous example (Bhalla and Proffitt, 1999) showed that people judge hills to be steeper when they are wearing a heavy backpack. The hypothesis is that the backpack would increase the effort required to climb the hill, and thus we perceive the hill as more difficult to climb (see Proffitt and Linkenauger, 2013 for a recent review).

Soliman et al. (2013) applied Proffitt's particular sensorimotor mechanism to a cultural context. They asked participants to judge the distances between themselves and in- or out-group members. The logic is as follows:

1. Interacting with someone requires you to (unconsciously) mentally simulate things they do
2. We do this simulation ahead of time, in anticipation of an encounter
3. This simulation will require more effort with out-group members, because they do things differently to you
4. This increased simulation effort will affect your judgment of distance, as per Proffitt
5. Therefore out-group members should look further away

The results seem promising; for example, American and Arab participants who rated themselves with an interdependent self-construal did rate their in-group members as closer than did participants with an independent self-construal. Soliman et al. argue that these results support an analysis of culture within Proffitt's embodied framework, which would be interesting if true because it would let us talk about both perceptual and cultural effects within a unified framework.

There is a major problem, however. Proffitt's research very clearly shows that increasing the effort required to perform a task only affects distance perception related to that task. For example, making walking harder by fatiguing the legs increases the perceived distance if you plan to cross it by walking, but not if you plan to cross it by throwing (Witt et al., 2010). Soliman et al., however, claim that the increased effort of internally simulating the movements required to interact with an out-group member will increase the perceived distance to that person when that distance is to be traversed by locomotion. Their effort manipulation has nothing to do with locomoting across the distance and thus, contrary to the framing of their paper, their results are neither predicted by nor explained by reference to Proffitt's action-scaling theory.

Task specificity is central to Proffitt's theory. In a recent debate, Firestone (2013) highlighted this because he believes this creates a problem for Proffitt; if distance perception for walking and throwing are calibrated to different scales, you cannot compare the two in order to choose the best way to cross that distance. Proffitt (2013) disagreed that this creates a problem but completely agreed that action-specific units are incommensurable in this way. He stated “An important finding across our studies is that the influence of an action unit—such as graspability—is evident only within its action boundary” (p. 477). This exchange is relevant because Proffitt is specifically challenged here on this point and comes out strongly and unambiguously in favor of task-specificity.

Soliman et al. rest their non-task-specific analysis on one paper (Schnall et al., 2008). Participants in this study judged hills as less steep when accompanied by or thinking about a friend. The claim here is that social support makes the hill appear more easily traversed, without any apparent recalibration of task-relevant effectors. Soliman et al. argue that this supports their hypothesis that an increase in upcoming social effort (the hypothesized prospective internal simulation of the other person described in points 1–3 above) could alter distance perception. However, it is worth noting that Schnall et al. specifically caution that “it is too early to speculate on the degree to which these influences [the effects of physical vs. psychological states on slant judgments] share common underlying mechanisms or on what these mechanisms might be” (p. 1254). In addition, when we place this single result in the context of the rest of Proffitt's extensive body of work repeatedly demonstrating strong task specificity, it actually seems more likely right now that the *only* way in which a friend could help make a hill look more climbable is by doing something that recalibrates the embodied hill-climbing system. Discovering what this something is would be an invaluable contribution to the unification Soliman et al. propose, but it remains to be done.

## Summary

Soliman et al. (2013) claim that the increased mental effort required to simulate an upcoming encounter with an out-group member will make the distance to that person look more difficult to cross and thus the person will look farther away. They ground this hypothesis in Proffitt's embodied action-scaling theory of perception, but Proffitt's data supports a strong form of task-specificity that means his theory neither predicts nor explains the current results.

The current data (plus Schnall et al., 2008) may eventually motivate a less task-specific version of Proffitt's mechanism. For example, the interaction of self-construal with distance Soliman et al. find is consistent with the claim that the interdependent and independent groups are evaluating the distances using different metrics (see p. 4–5). But whether those metrics are effort based (overturning the otherwise extensive evidence in favor of task-specificity) remains to be confirmed.

I am personally all in favor of an embodied approach to unifying psychology, but as I have argued (Wilson and Golonka, 2013) this will require careful attention to the details of the relevant sensorimotor (perception-action) mechanisms so that we are sure we are connecting them to “higher level” cognition in ways that reflect how those mechanisms actually operate. This connection is simply not present in the target article, and the implication for Soliman et al. is that their data do not support their particular attempt to unify psychology with sensorimotor mechanisms.

### Conflict of interest statement

The author declares that the research was conducted in the absence of any commercial or financial relationships that could be construed as a potential conflict of interest.

## References

[B1] Bhalla M. Proffitt D. R. (1999). Visual-motor recalibration in geographical slant perception. J. Exp. Psychol. Hum. Percept. Perform. 25, 1076–1096 10.1037//0096-1523.25.4.1076 10464946

[B2] Firestone C. (2013). How “paternalistic” is spatial perception? Why wearing a heavy backpack doesn't - and couldn't - make hills look steeper. Perspect. Psychol. Sci. 8, 455–473 10.1177/1745691613489835 26173123

[B3] Gibson J. J. (1966). The Senses Considered as Perceptual Systems. Boston, MA: Houghton-Mifflin

[B4] Gibson J. J. (1979). The Ecological Approach to Visual Perception. Boston, MA: Psychology Press

[B5] Proffitt D. R. (2013). An embodied approach to perception: by what units are visual perceptions scaled? Perspect. Psychol. Sci. 8, 474–483 10.1177/1745691613489837 26173124

[B6] Proffitt D. R. Linkenauger S. A. (2013). Perception viewed as a phenotypic expression, in Action Science: Foundations of an Emerging Discipline, eds Prinz W. Beisert M. Herwig A. (Cambridge, MA: MIT Press), 171–197

[B7] Schnall S. Harber K. Stefanucci J. Proffitt D. R. (2008). Social support and the perception of geographical slant. J. Exp. Soc. Psychol. 44, 1246–1255 10.1016/j.jesp.2008.04.011 22389520 PMC3291107

[B8] Soliman T. Gibson A. Glenberg A. M. (2013). Sensory motor mechanisms unify psychology: the embodiment of culture. Front. Psychol. 4:885 10.3389/fpsyg.2013.00885 24348439 PMC3843157

[B9] Wilson A. D. Golonka S. (2013). Embodied cognition is not what you think it is. Front. Psychol. 4:58 10.3389/fpsyg.2013.00058 23408669 PMC3569617

[B10] Witt J. K. Proffitt D. R. Epstein W. (2010). When and how are spatial perceptions scaled? J. Exp. Psychol. Hum. Percept. Perform. 36, 1153–1160 10.1037/a0019947 20731519

